# Giant proximity effect in single-crystalline MgB_2_ bilayers

**DOI:** 10.1038/s41598-019-40263-9

**Published:** 2019-03-01

**Authors:** Soon-Gil Jung, Duong Pham, Tae-Ho Park, Han-Yong Choi, Jin Won Seo, Won Nam Kang, Tuson Park

**Affiliations:** 1 Center for Quantum Materials and Superconductivity (CQMS), Sungkyunkwan University, Suwon, 16419 Republic of Korea; 2 Department of Physics, Sungkyunkwan University, Suwon, 16419 Republic of Korea; 3 Department of Materials Engineering, KU Leuven, Kasteelpark Arenberg 44-bus 2450, B-3001 Leuven, Belgium

## Abstract

Although giant proximity effect (GPE) can shed important information on understanding superconducting pairing mechanisms and superconducting electronics, reports on the GPE are few because the fabrication of the junctions with GPE is technologically difficult. Here, we report a GPE in the single-crystalline MgB_2_ bilayers (*S*′/*S*), where the *S*′ is the damaged MgB_2_ layer by cobalt (Co)-ion irradiation and the *S* is the undamaged MgB_2_ layer. Superconducting properties of the *S*′ is remarkably degraded by the irradiation, whereas those of the *S* is uninfluenced by the irradiation. The degraded superconductivity in the *S*′ is fully recovered by increasing the thickness of undamaged MgB_2_ layer *S* despite almost ten times larger thickness ~ 95 nm of *S*′ than the superconducting coherence length *ξ*_ab_(0) ~ 8.5 nm of the *S*, indicating a presence of GPE in the *S*′*/S* MgB_2_ bilayers. A diffusion of electrons in the *S*′ into the *S* can reduce a pair breaking scattering in the *S*′, and the similar electronic structures of *S*′ and *S* layers and a finite attractive electron-electron interaction in the *S*′ are thought to be origins of unpredicted GPE between the same superconducting materials. Both upper critical field (*μ*_0_*H*_c2_) and in-field critical current density (*J*_c_) of *S*′/*S* bilayers show a significant enhancement, representing a strong correlation between *S*′ and *S*. These discoveries provide the blue print to the design of the superconducting multilayers for fundamental researches on the mechanism of the GPE as well as their technological applications.

## Introduction

The superconducting proximity effect (PE), a leak of Cooper pairs from a superconductor (*S*) into a normal metal (*N*) when they are in contact with each other, is a fascinating phenomenon that is critical to the design of superconducting electronic devices, such as superconducting quantum interface device (SQUID) and quantum information device^1–4^. Superconducting coherence length (*ξ*) and electronic mean free path (*l*) in the normal metal are important parameters to determine the leaking distance of Cooper pairs in *S/N* junctions, which have been intensively studied and well understood based on conventional theories^2,3,5–10^.

Recently, unpredicted large PE was observed when superconductor *S*_1_ is connected with another superconductor *S*_2_ instead of normal metal, which is known as the giant proximity effect (GPE)^11–17^. Here, superconducting transition temperature (*T*_c_) of *S*_2_ is lower than that of *S*_1_. The Cooper-pair leaking distance in *S*_1_/*S*_2_ junctions is almost ten times larger than the coherence length *ξ*, and Josephson critical current could be much improved in the superconducting multilayers composed of the optimally doped and under doped high-*T*_c_ cuprates, such as LSCO (La_2-*x*_Sr_*x*_CuO_4_)/LCO (La_2_CuO_4+*δ*_)/LSCO trilayer^13–15^. The spatially long-ranged propagation of Cooper pairs between two different conventional superconductors, such as few-layer lead (Pb) island and Pb monolayer, was also visualized by using scanning tunnelling spectroscopy (STS) and scanning tunnelling microscopy (STM)^16,17^. The Cooper pairs’ leaking distance is influenced by the *T*_c_ of *S*_2_, which is considered due to its finite attractive interactions/phase fluctuations or an additional superconductivity between the interface of *S*_1_ and *S*_2_. However, the origin of GPE has yet to be clarified^12–15,18^.

GPE is expected to provide a significant technological advantage for the applications of superconducting electronics because thicker barrier can make it much easier to achieve uniform Josephson junctions. GPE observed in the cuprates-based Josephson junctions, however, has shown inconsistent results partly because of the surface roughness between layers and secondary phases^13,15^. Fabrication of the uniform multilayers in such complex compounds has proven to be difficult so far.

Ion irradiations have been often used to fabricate *S/N/S* Josephson junctions because local crystal disorders or defects can easily be controlled by an irradiating ion source or its dose^19–23^, and long range PE has been also observed in the junctions fabricated by ion irradiations^23^. Similar electronic structures between *S* and *N* layers and a finite SC pairing interaction in the *N* layer are considered as sources for the enhanced proximity effect^13,14,16,24^. Conventional superconductor MgB_2_ with relatively high *T*_c_ of 40 K is a good candidate for GPE realization via the ion irradiation technique because of its large superconducting coherence length, metallicity, and large superconducting energy gap^25,26^.

Here we report the giant proximity effect in the *S*′/*S* MgB_2_ bilayers. A dose of 1 × 10^14^ Co atoms/cm^2^ with the 140 keV beam energy (mean projected range *δ* = 95 nm), where *δ* is the average distance from the surface of the film at which the irradiated Co ions come to maximum concentration, is irradiated into single-crystalline MgB_2_ films with various thicknesses (*t*) of 130, 200, 410, 850, and 1,300 nm. *T*_c_ of the MgB_2_ film with *t* = 130 nm is considerably suppressed from 38.2 to 4.5 K after the Co-ion irradiation. Although, the thickness of damaged MgB_2_ layer (*S*′) by the irradiation is almost ten times longer than the coherence length, *ξ*_ab_(0) ~ 8.5 nm, of the undamaged MgB_2_ layer (*S*), the suppressed *T*_c_ of *S*′ by the irradiation is rapidly restored to that of the pristine state with an increase in the thickness of *S*. In addition, upper critical field (*μ*_0_*H*_c2_) of the *S*′/*S* bilayers has two times larger value than that of the pristine films, and field performance of critical current density (*J*_c_) of the bilayers is superior to that of the pristine MgB_2_ films. These results demonstrate that the existence of giant proximity effect in the *S*′/*S* MgB_2_ bilayers provides a blue print to fabricate superconducting MgB_2_ multilayers for their fundamental researches as well as technological applications.

## Results

**Figure 1:**
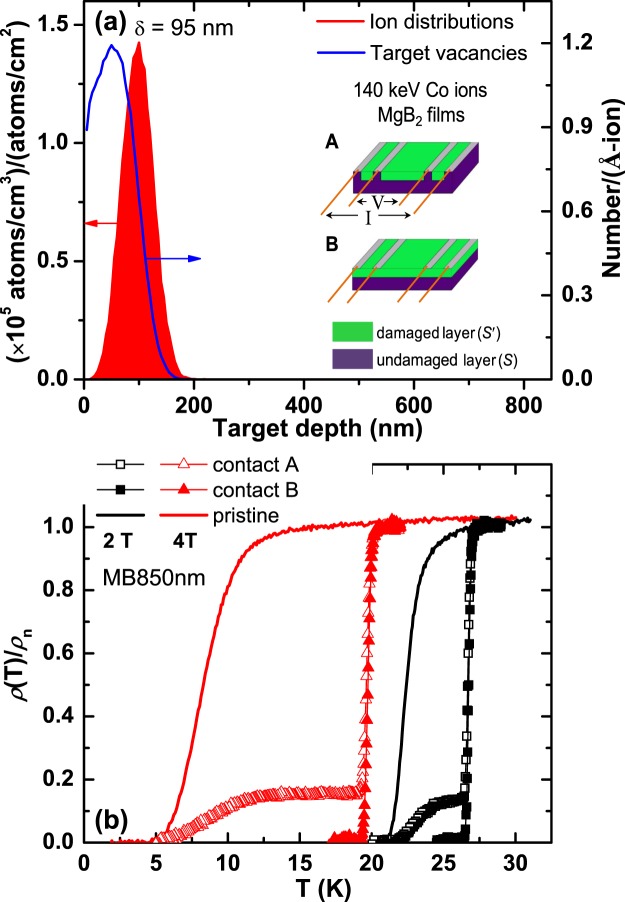
Schematic view of MgB_2_/MgB_2_ bilayers fabricated by cobalt ion irradiation. (**a**) SRIM simulation for the mean projected range (*δ*) and target vacancies resulted from damaged events of irradiated Co ions with an incident energy of 140 keV in MgB_2_, where the estimated *δ* is around 95 nm. Insets show two types of configurations for the four-probe point contacts: contact method A and B. Electrical resistivity (*ρ*) measured by the contact method A is influenced by both damaged layer (*S*′) and undamaged layer (*S*). On the other hand, the SC transition measured by the contact method B is determined by the *S*′. (**b**) Temperature dependences of the normalized resistivity, *ρ*(*T*)/*ρ*_n_, for the pristine and irradiated MB850 nm at magnetic fields of 2 and 4 T, where *ρ*_n_ is the resistivity at *T*_c_ onset at each magnetic field. The kinks and the second SC transitions observed from the contact method A are due to the undamaged layer *S*, indicating the formation of the *S*′*/S* MgB_2_ bilayer via the Co-ion irradiation.

Figure 1(a) shows the mean projected range (*δ* = 95 nm) of the irradiated 140 keV Co ions into MgB_2_ and damage events created by the irradiated energetic ions, simulated by the SRIM program^27^. If MgB_2_ film is thick enough compared to the *δ*, it can be divided into two layers: damaged layer (*S*′) and undamaged layer (*S*). Superconducting (SC) properties of the *S* is expected to be same with that of the pristine state, while the SC properties of the *S*′ will be changed by the ion-irradiation conditions. In order to investigate the correlation between *S*′ and *S*, we considered two types of electrode configurations, i.e., 4-point contact method A and B, as described in the insets of Fig. 1(a). In contact method A, the 4 probes are directly connected to the *S*, whereas they are in contact with the *S*′ in contact method B.

The solid lines in Fig. 1(b) indicate the temperature dependences of electrical resistivity (*ρ*) for the pristine MB850 nm at magnetic fields of 2 and 4 T, where *ρ*(*T*) is normalized by the *ρ* value at *T*_c_ onset, *ρ*_n_, at each magnetic field for comparison. After irradiation with a dose of 1 × 10^14^ Co atoms/cm^2^ in the MB850 nm, *ρ*(*T*) was measured again by using the 4-point contact method A and B. For the contact method B, the resistivity drop of the irradiated film at *T*_c_ is sharper and more robust than that of the pristine: at 4 Tesla, the zero-resistance state is suppressed to 19.1 K for the irradiated film, while it is 4.8 K for the pristine. The *ρ*(*T*) curves measured by the contact method A, in contrast, show two SC transitions: the first SC transition is consistent with the SC transition of the *S*′ measured by the contact method B and the second SC transition is similar to the SC transition of the pristine sample. These results delineate that *S*′*/S* MgB_2_ bilayer is fabricated via the ion irradiation method.

**Figure 2:**
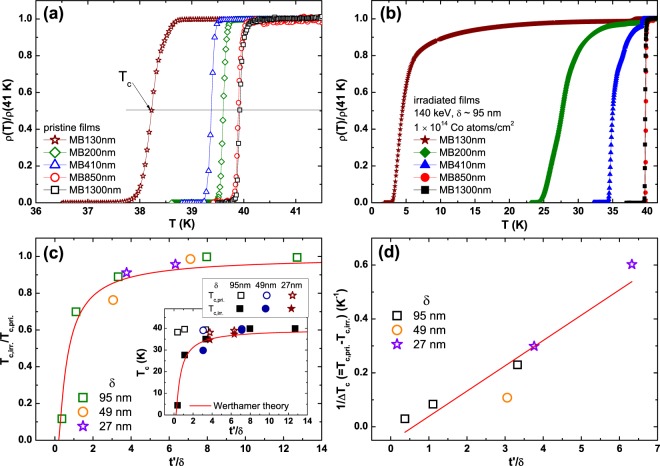
Thickness dependence of superconducting transition temperature for irradiated MgB_2_ films. *ρ*(*T*) curves near the SC transitions for (**a**) pristine and (**b**) Co-ion irradiated MB130 nm, MB200 nm, MB410 nm, MB850 nm, and MB1300 nm, where *ρ*(*T*) was normalized by the *ρ* at 41 K, *ρ*(41 K), for comparison. (**c**) The SC transition temperature of the irradiated films (*T*_c,irr._) as a function of *t*′*/δ*, where the *T*_c,irr._ is normalized by *T*_c_ of the pristine films (*T*_c,pri._) and *t*′ (=*t* − *δ*) is the thickness of the *S*. Star, circle, and square symbols represent *T*_c_s for different incident energies of 35 (*δ* ~ 27 nm), 70 (*δ* ~ 49 nm), and 140 keV (*δ* ~ 95 nm), respectively. Both *T*_c,pri._ and *T*_c,irr._ are determined from the midpoint of SC transition, *ρ*_50%_, 50% resistivity drop from the *ρ*_n_. The red solid line is a fitting curve by using the Werthamer theory. Inset shows the *T*_c,pri._ and *T*_c,irr._ as a function of *t*′*/δ*. (**d**) The inverse of the *T*_c_ reduction, 1/Δ*T*_c_ (=*T*_c,pri._ − *T*_c,irr._), is plotted as a function of *t*′*/δ*.

The *ρ*(*T*) curve near *T*_c_ for pristine MgB_2_ films of MB130 nm, MB200 nm, MB410 nm, MB850 nm, and MB1300 nm is shown in Fig. 2(a), where *ρ*(*T*) is normalized by the *ρ* value at 41 K, *ρ*(41 K). With decreasing film thickness, *T*_c_ of the pristine MgB_2_ films slightly decreases and the SC transition remains sharp, except the thinnest MB130 nm (ref.^28^) (see Fig. S1 in the Supplemental Information). On the other hand, *T*_c_ of the *S*′*/S* MgB_2_ bilayers is rapidly suppressed with decreasing the thickness (*t*′) of undamaged MgB_2_ layer *S*, as shown in Fig. 2(b), where *ρ*(*T*) was measured by using the contact method A. The change in *T*_c_ of the *S*′*/S* bilayers is linked to *t*′ because the same dose of Co ions with the same incident energy of 140 keV was irradiated. The large suppression of *T*_c_ from 38.2 to 4.5 K in MB130 nm after the irradiation indicates that a dose of 1 × 10^14^ Co atoms/cm^2^ is enough to substantially degrade superconductivity of the MgB_2_. Interestingly, however, *T*_c_ is rapidly restored back to the original value with increasing film thickness. Temperature dependences of dc magnetization (*M*) also showed similar results to the *ρ*(*T*) curve (Fig. S2 in the Supplemental Information), indicating that the SC transition in the *S*′/*S* bilayers is not originated from filamentary nature. In addition, the fact that the thickness of damaged MgB_2_ layer (*S*′) is much larger than the coherence length (*ξ*) of MgB_2_ (ref.^26^) indicates the emergence of giant proximity effect (GPE) in *S*′*/S* MgB_2_ bilayers. We note that the GPE is often observed in *S*_1_/*S*_2_ junctions, where *S*_1_ and *S*_2_ are superconducting layers with different *T*_c_ (refs^11–17^).

Figure 2(c) displays the SC transition temperature of the irradiated MgB_2_ films (*T*_c,irr._), normalized by that of the pristine films (*T*_c,pri._), as a function of the reduced film thickness *t*′*/δ*, where *t*′ (=*t − δ*) represents the thickness of the undamaged layer (*S*) and *δ* corresponds to the thickness of the damaged layer (*S*′). Here, *T*_c,pri._ and *T*_c,irr._ are determined from the criterion of 50% resistivity drop from the resistivity value at 41 K. For comparison, *T*_c,irr._/*T*_c,pri._ for *S*′/*S* bilayers with *δ* = 27 (stars) and 49 nm (circles) are also plotted in Fig. 2(c), where a beam energy of 35 (*δ* ~ 27.3 nm) and 70 keV (*δ* ~ 49.4 nm) was used with the same dose of 1 × 10^14^ Co atoms/cm^2^. Inset of Fig. 2(c) shows *T*_c,pri._ and *T*_c,irr._ as a function of the *t*′/*δ* for pristine and irradiated MgB_2_ films, respectively. *T*_c,irr._ that corresponds to the *T*_c_ of *S*′*/S* bilayers is monotonically reduced with decreasing *t*′/*δ* and is completely suppressed to 0 K at the critical ratio *t*′/*δ* ~ 0.19, i.e. 19% of the thickness of *S* to that of *S*′. For *δ* = 95 nm, for instance, the critical thickness (*T*_c,irr._ = 0 K) of the *S*′*/S* MgB_2_ bilayer is 113 nm.

## Discussion

The thickness ratio (*t*′*/δ*) dependence of the SC transition temperature for the *S*′*/S* bilayers is expressed by the Werthamer theory considered the spatial variation of electron-electron interaction^6^, even though the thicknesses of the *S* and *S*′ are much thicker than coherence length of MgB_2_. In the BCS theory, *T*_c_ of a normal metal (*N*) and superconductor (*S*) bilayer can simply be expressed by the relation *T*_c_ = 1.14*Θ*_D_exp[−1/(*N*_0_*V*_0_)_eff_], where *Θ*_D_ is Debye temperature, *N*_0_ is the electronic density of states at the Fermi energy, and *V*_0_ is strength of attractive electron-electron interaction, and (*N*_0_*V*_0_)_eff_ is the effective BCS interaction parameter^2,6,29^. For *N/S* bilayer, *N*_0_*V*_0_ in the *S* layer is an important parameter to determine *T*_c_ of the bilayer, because *V*_0_ = 0 in the *N* layer. On the other hand, a finite *V*_0_ in the *N* layer could induce the long range of the PE due to the slow decay of SC pair amplitude in the *N* layer^16,18,24^, which is probably one of the primary reasons for GPE in the *S*′*/S* MgB_2_ bilayer.

The electronic density of states at the Fermi energy (*N*_0_) is also important to determine *T*_c_, and the large reduction of *T*_c_ in MgB_2_ by the irradiation is believed to be due to the decreased *N*_0_ and enhanced interband scattering^30–34^. Considering a significant decrease of *T*_c_ of MB130 nm from 38.2 to 4.5 K by Co-ion irradiation, the *T*_c_ variation in the *S*′*/S* MgB_2_ bilayers is expressed by the decrease in the *N*_0_ (refs^29,35,36^). Figure 2(d) shows that the inverse of *T*_c_ difference between the pristine and irradiated films, 1/Δ*T*_c_ (=*T*_c,pri._ − *T*_c,irr._), is linearly proportional to the thickness ratio between the undamaged (*t*′) and damaged MgB_2_ layers (*δ*), *t*′/*δ*, indicating that the *T*_c_ suppression by the Co-ion irradiation is closely related with the decrease in the electronic density of states *N*_0_ of the damaged layer *S*′ (refs^29,35,36^). The initial slope of *T*_c_ (∝|*dT*_c_(*d*_n_ = 0)/*dd*_n_|) in *S/N* bilayer is proportional to the ratio of density of states between the two layers (*N*_n_/*N*_s_) because *T*_c_ = 1.14 *Θ*_D_exp[−1/(*N*_0_*V*_0_)], where *N*_n_ and *N*_s_ are the density of states at the Fermi energy for the normal and superconducting layer, respectively, and *d*_n_ is a thickness of the normal layer^29,35,36^. Our results on the *T*_c_ reduction with respect to the thickness ratio *t*′/*δ* is consistent with the decrease in the density of states of the *S*′ caused by the irradiation-induced disorder^30–34^.

We consider the diffusion of electrons inside the damaged *S*′ into the undamaged *S* as well as the diffusion of Cooper pairs from the *S* to *S*′. Those diffusion processes by the electrons and the Cooper pairs can lead the extension of the proximity region in terms of the strong correlation between *S* and *S*′. In particular, when the thickness of *S* is large, there can be enough space for the diffusion of electrons from the *S*′ into *S*. This interpretation is supported by the study on the lower and upper critical fields and critical current density for *S*′*/S* MgB_2_ bilayers, which will be discussed below.

**Figure 3:**
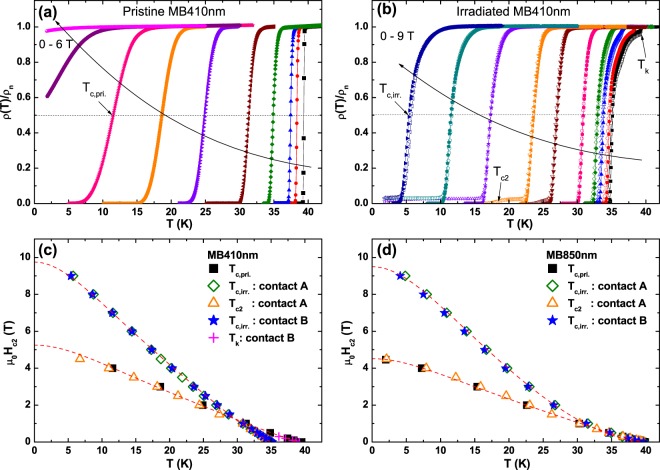
Upper critical fields of MgB_2_/MgB_2_ bilayers. *ρ*(*T*) curves for (**a**) pristine and (**b**) Co-ion irradiated MB410 nm in magnetic fields, where *ρ*(*T*) was normalized by the *ρ*_n_ at each magnetic field. In (**b**), the open and solid symbols are obtained from the contact method A and B, respectively. Temperature dependence of upper critical field, *μ*_0_*H*_c2_(*T*), for (**c**) MB410 nm and (**d**) MB850 nm. Criteria for *T*_c,pri._, *T*_c,irr._, *T*_c2_, and *T*_k_ are indicated by arrows in (**a,b**). The red dashed lines are obtained from the two-band Ginzburg-Landau theory. The *T*_c2_’s obtained from the second SC transition are consistent with *T*_c,pri._, demonstrating that the irradiated films are composed of the *S*′*/S* bilayer: damaged MgB_2_ layer (*S*′) and undamaged MgB_2_ layer (*S*).

Figure 3(a,b) show the *ρ*(*T*) curves in magnetic fields for the pristine and Co-ion irradiated MB410 nm, respectively, where the *ρ*(*T*) is normalized by the resistivity value in the normal state (*ρ*_n_) for each magnetic field. In stark contrast to the pristine film, two-step SC transitions appear in the *ρ*(*T,H*) measured by the 4-probe contact method A for the Co-ion irradiated MB410 nm. The second SC transition temperature (*T*_c2_) is consistent with the SC transition temperature of pristine films (*T*_c,pri._), indicating that *T*_c2_ emerged at high magnetic fields is originated from superconductivity in the *S*. When the contact method B was used, on the other hand, there is no trace of *T*_c2_ in *ρ*(*T,H*) curves because the 4 probes of the contact method B are not directly in contact with the undamaged layer and the supercurrent flows mainly through the damaged layer *S*′.

Temperature dependences of the upper critical field (*μ*_0_*H*_c2_) for the pristine and irradiated MB410 nm and MB850 nm are presented in Fig. 3(c,d), respectively, where the magnetic field is applied perpendicular to the film’s *ab* plane (*H* ⊥ *ab*). The criteria for *T*_c,pri._ and *T*_c,irr._ are 50% resistivity drop for the pristine and irradiated films, respectively, while *T*_c2_, and *T*_k_ are assigned as the 50% resistivity drop of the second SC transition and a kink temperature of the first SC transition, respectively (see the arrows in Fig. 3(a,b)). The magnetic field dependence of the kink temperature (*T*_k_) for the MB410 nm suggests that *T*_k_ is associated with *T*_c_ of the undamaged layer *S*. One the other hand, there is no kink (*T*_k_) in the irradiated MB850 nm because superconductivity of the damaged layer *S*′ is completely restored to that of the pristine state, i.e., *T*_c,pri._ ≈ *T*_c,irr._. The fact that *μ*_0_*H*_c2_(*T*)s determined by *T*_c2_ are consistent with those estimated from the *T*_c,pri._ underscores that the MgB_2_/MgB_2_ bilayer is formed by the Co-ion irradiation. Upper critical field of the bilayers is enhanced by two times to the pristine films: *μ*_0_*H*_c2_(0) = 5.29 → 9.75 T for MB410 nm and *μ*_0_*H*_c2_(0) = 4.52 → 9.50 T for MB850 nm, which can be accounted for by additional defects introduced via the Co-ion irradiation^37^. The SC coherence length, *ξ*_ab_(0), obtained from the *μ*_0_*H*_c2_(0) [=*ϕ*_0_/2*πξ*^2^_ab_(0)] is reduced from 7.89 to 5.81 nm and from 8.54 to 5.89 nm for MB410 nm and MB850 nm after the irradiation, respectively.

Temperature dependence of *μ*_0_*H*_c2_ is reasonably explained by the two-band Ginzburg-Landau theory, indicated by the red dashed lines in Fig. 3(c,d). According to Takahashi-Tachiki model in *S*_1_/*S*_2_ superlattices, an upturn behavior in *H*_c2_(*T*) can be observed near *T*_c_ due to different spatial variations in the density of states, the diffusion constant, and the electron-electron attractive interaction potential between *S*_1_ and *S*_2_ (refs^38,39^). The enhanced upturn feature of *μ*_0_*H*_c2_(*T*) in the MgB_2_ bilayer (MB850 nm) compared to that in the pristine film may be closely associated with the different diffusion constant between *S* and *S*′ layers because *T*_c,pri._ ≈ *T*_c,irr._ in the MB850 nm. The Cooper pairs in the damaged layer may be more effective in penetrating into the undamaged layer, inverse proximity effect^16,23^, than the reversal process because of a larger diffusion constant in the *S* than *S*′ layer, leading to the large upturn feature in *μ*_0_*H*_c2_(*T*) near *T*_c_. On the other hand, Ferrando *et al*. suggested that a large upward behavior of *H*_c2_(*T*) near *T*_c_ in MgB_2_ thin films is associated with a large resistive surface layer of MgB_2_ due to the contamination in air rather than the multiband effect of MgB_2_ (refs^40,41^). In order to confirm this mechanism, however, further measurements for *μ*_0_*H*_c2_(*T*) in high magnetic field parallel to the *ab* plane (*H//ab*) will be needed because this upturn behavior or sudden change of *μ*_0_*H*_c2_(*T*) in *S*′*/S* is more prominent for *H//ab* (refs^38,39^).

**Figure 4:**
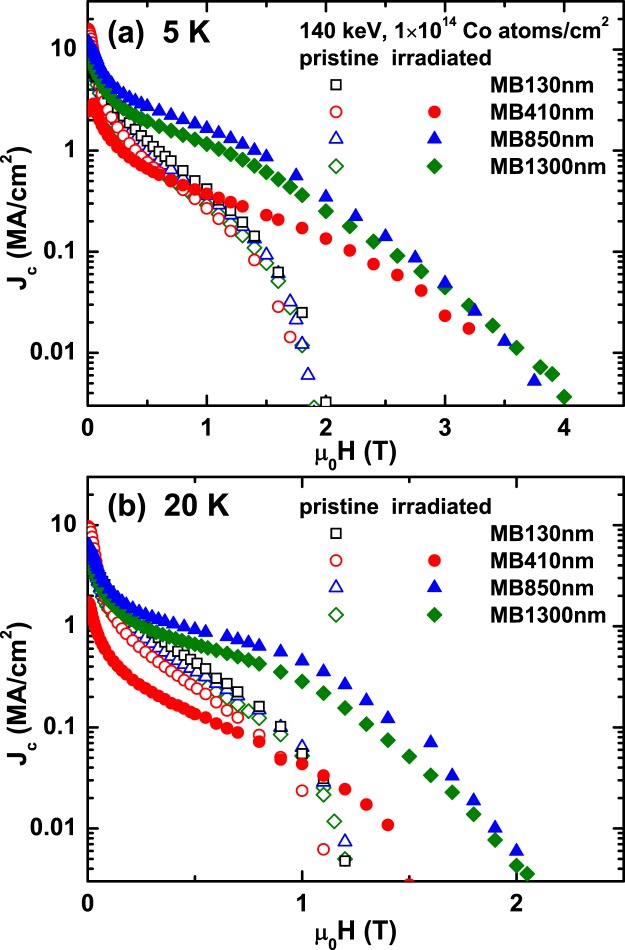
Critical current density, *J*_c_, of MgB_2_/MgB_2_ bilayer. Magnetic field dependences of the critical current density (*J*_c_) at (**a**) 5 and (**b**) 20 K for the pristine and Co-ion irradiated MB130 nm, MB410 nm, MB850 nm, and MB1300 nm. *J*_c_ for all pristine films shows a similar field performance, and a rapid decrease of *J*_c_ in magnetic fields indicates that the MgB_2_ films used in this study is of high quality. A significant enhancement of in-field *J*_c_ after irradiation is observed except for the MB130 nm. There is no data for the *J*_c_ of the irradiated MB130 nm due to a substantially suppressed *T*_c_ and SC volume fraction.

Magnetic field dependences of critical current density (*J*_c_) for the pristine and irradiated MgB_2_ films at 5 and 20 K are shown in Fig. 4(a,b), respectively, where the magnetic field is applied perpendicular to the film’s *ab* plane. The large *J*_c_ at zero field and its rapid suppression in magnetic fields indicate that the pristine MgB_2_ films used in this study is clean^42,43^. After Co-ion irradiation, in-field *J*_c_ is significantly improved except for the thinnest MB130 nm film because of the large suppression of *T*_c_. The suppression of zero-field *J*_c_ for the irradiated MB410 nm is also related with the suppressed *T*_c_ by the irradiation. On the other hand, in-field *J*_c_ for both MB850 nm and MB1300 nm is remarkably enhanced without the suppression of the zero-filed *J*_c_ after Co-ion irradiation, suggesting that the proximity effect (PE) in the *S*′*/S* bilayers is not confined near the interface^11–13^, but is long ranged over the whole thickness of the irradiated layer *S*′ when the thickness of the *S* is sufficiently larger than that of the *S*′. In addition, the large in-field *J*_c_ in *S*′*/S* MgB_2_ bilayers manifests the strong correlation between *S*′ and *S* layers.

## Conclusion

In conclusion, we reported fabrication of MgB_2_/MgB_2_ bilayers, where a dose of 1 × 10^14^ Co atoms/cm^2^ with a beam energy of 140 keV is irradiated into single-crystalline MgB_2_ films and created the damaged layer (*S*′) interfaced with the undamaged layer (*S*). Superconductivity of MgB_2_ in the damaged layer is significantly weakened: *T*_c_ is suppressed from 38.2 to 4.5 K for the irradiated MB130 nm film. The suppressed superconductivity, however, was restored to the original SC state when the thickness of the undamaged MgB_2_ layer (*S*) is sufficiently larger than that of the damaged MgB_2_ layer (*S*′). Although the coherence length of the undamaged layer *S* (~8.5 nm) is more than ten times shorter than the damaged layer *S*′ (~95 nm), the proximity effect occurs over the whole thickness of the *S*′. This anomalously long-ranged proximity effect in *S*′/*S* MgB_2_ bilayers is thought to be originated from the similar electronic structures of *S*′ and *S* layers and a finite SC pairing interaction in the *S*′ layer. The discovery of giant proximity effect (GPE) in the MgB_2_/MgB_2_ bilayers is expected to provide a blue print to the design of superconducting MgB_2_ multilayers for their fundamental researches as well as technological applications.

## Methods

Hybrid physical-chemical vapour deposition (HPCVD) method was used for the growth of single-crystalline MgB_2_ films on the *c*-cut Al_2_O_3_ substrates, and the details of the growth technique and the film’s single-crystal quality are described elsewhere^43,44^. The MgB_2_ films with various thicknesses (*t*) of 130 (MB130 nm), 200 (MB200 nm), 410 (MB410 nm), 850 (MB850 nm), and 1,300 nm (MB1300 nm) were fabricated for ion irradiations, and the same amount of dose of 1 × 10^14^ Co atoms/cm^2^ with 140 keV beam energy at room temperature was irradiated into the prepared MgB_2_ films in Korea Multi-purpose Accelerator Complex (KOMAC). The mean projected range (*δ*) of irradiated Co ions is around 95 nm, which is simulated by the Monte Carlo simulation program SRIM (The Stopping and Range of Ions in Matter)^27^.

The formation of *S*′/*S* MgB_2_ bilayers by the Co-ion irradiation was confirmed by simultaneously measuring electrical resistivity (*ρ*) of the damaged and undamaged MgB_2_ layers in Physical Property Measurement System (PPMS 9 T, Quantum Design). The standard 4-probe method was used for electrical resistivity measurements, and the 4-point contact regions were protected from the irradiation by using gold (Au) coating and silver (Ag) epoxy in order to measure the resistivity of the undamaged layer after the irradiation. In order to estimate the critical current density (*J*_c_), magnetization hysteresis (*M – H*) loops were measured by using a Magnetic Property Measurement System (MPMS 5 T, Quantum Design) before and after the Co-ion irradiations (see Fig. S3 in the Supplemental Information).

## Supplementary information

Supplementary Information

## References

[1] Meissner H (1960). Superconductivity of contacts with interposed barriers. Phys. Rev..

[2] de Gennes PG (1964). Boundary effects in superconductors. Rev. Mod. Phys..

[3] Likharev KK (1979). Superconducting weak links. Rev. Mod. Phys..

[4] Makhlin Y, Schön G, Shnirman A (2001). Quantum-state engineering with Josephson-junction devices. Rev. Mod. Phys..

[5] Hilsch P, Hilsch R (1964). Zur Supraleitung von Schichtpaketen aus Normal- und Supraleitern. Z. Phys..

[6] Werthamer NR (1963). Theory of the superconducting transition temperature and energy gap function of superposed metal films. Phys. Rev..

[7] Kircher CJ (1968). Superconducting proximity effect of Nb. Phys. Rev..

[8] Hauser JJ, Theuerer HC, Werthamer NR (1964). Superconductivity in Cu and Pt by means of superimposed films with lead. Phys. Rev..

[9] de Gennes PG, Guyon E (1963). Superconductivity in “normal” metals. Phys. Lett..

[10] Kim J, Doh Y-J, Char K, Doh H, Choi H-Y (2005). Proximity effect in Nb/Au/CoFe trilayers. Phys. Rev. B.

[11] Decca RS, Drew HD, Osquiguil E, Maiorov B, Guimpel J (2000). Anomalous proximity effect in underdoped YBa_2_Cu_3_O_6+*x*_ Josephson junctions. Phys. Rev. Lett..

[12] Marchand D, Covaci L, Berciu M, Franz M (2008). Giant proximity effect in a phase-fluctuating superconductor. Phys. Rev. Lett..

[13] Bozovic I (2004). Giant proximity effect in cuprate superconductors. Phys. Rev. Lett..

[14] Covaci L, Marsiglio F (2006). Proximity effect and Josephson current in clean strong/weak/strong superconducting trilayers. Phys. Rev. B.

[15] Rout PK, Budhani RC (2010). Interface superconductivity in La_1.48_Nd_0.4_Sr_0.12_CuO_4_/La_1.84_Sr_0.16_CuO_4_ bilayers. Phys. Rev. B.

[16] Cherkez V (2014). Proximity effect between two superconductors spatially resolved by scanning tunneling spectroscopy. Phys. Rev. X.

[17] Kim J (2012). Visualization of geometric influences on proximity effects in heterogeneous superconductor thin films. Nat. Phys..

[18] Quintanilla J, Capelle K, Oliveira LN (2003). Comment on “Anomalous proximity effect in underdoped YBa_2_Cu_3_O_6+*x*_ Josephson junctions”. Phys. Rev. Lett..

[19] Kahlmann F (1998). Superconductor–normal–superconductor Josephson junctions fabricated by oxygen implantation into YBa_2_Cu_3_O_7-δ_. J. Appl. Phys. Lett..

[20] Cybart SA (2006). Planar MgB_2_ Josephson junctions and series arrays via nanolithography and ion damage. Appl. Phys. Lett..

[21] Kang D-J (2002). Realization and properties of YBa_2_Cu_3_O_7−δ_ Josephson junctions by metal masked ion damage technique. Appl. Phys. Lett..

[22] Sirena M (2009). Annealing of ion irradiated high *T*_c_ Josephson junctions studied by numerical simulations. J. Appl. Phys..

[23] Sharafiev A (2018). HTS Josephson junctions arrays for high-frequency mixing. Supercond. Sci. Technol..

[24] Tachiki M, Takahashi S (1992). Proximity effect in high-T_*c*_ oxide superconductors. Physics C.

[25] Cybart SA (2014). Large scale two-dimensional arrays of magnesium diboride superconducting quantum interference devices. Appl. Phys. Lett..

[26] Galan E, Cunnane D, Xi XX, Chen K (2014). Sandwich-type MgB_2_/TiB_2_/MgB_2_ Josephson junctions. Supercond. Sci. Technol..

[27] *The projected range of Co ions in the MgB*_*2*_*film was calculated using the SRIM software*. (www.srim.org/).

[28] Pogrebnyakov AV (2003). Thickness dependence of the properties of epitaxial thin films grown by hybrid physical-chemical vapor deposition. Appl. Phys. Lett..

[29] Zhang M, Tateishi G, Bergmann G (2006). Discrepancies between experiment and theory in the superconducting proximity effect. Phys. Rev. B.

[30] Ferrando V (2007). Neutron irradiation effects on two gaps in MgB_2_. Physica C.

[31] Gerashenko AP, Mikhalev KN, Verkhovskii SV, Karkin AE, Goshchitskii BN (2002). Reduction in the electron density of states in superconducting MgB_2_ disordered by neutron irradiation: ^11^B and ^25^Mg NMR estimates. Phys. Rev. B.

[32] Vinod K, Varghese N, Syamaprasad U (2007). Superconductivity of MgB_2_ in the BCS framework with emphasis on extrinsic effects on critical temperature. Supercond. Sci. Technol..

[33] Putti M, Vaglio R, Rowell JM (2008). Radiation effects on MgB_2_: a review and a comparison with A15 superconductors. Supercond. Sci. Technol..

[34] Putti M (2007). Intraband vs. interband scattering rate effects in neutron irradiated MgB_2_. EPL.

[35] Bergmann G (2005). Quantitative simulation of the superconducting proximity effect. Phys. Rev. B.

[36] Garrett D, Zhang M, Bergmann G (2004). The superconducting proximity effect as a tool to investigate metal films and interfaces. Eur. Phys. J. B.

[37] Tinkham, M. *Introduction to Superconductivity*, 2nd ed (McGraw- Hill, New York, 1996).

[38] Takahashi S, Tachiki M (1986). Theory of the upper critical field of superconducting superlattices. Phys. Rev. B.

[39] Takahashi S, Tachiki M (1986). New phase diagram in superconducting superlattices. Phys. Rev. B.

[40] Ferrando V (2007). Systematic study of disorder induced by neutron irradiation in MgB_2_ thin films. J. Appl. Phys..

[41] Gurevich A (2003). Enhancement of the upper critical field by nonmagnetic impurities in dirty two-gap superconductors. Phys. Rev. B.

[42] Hanna M (2008). Clean epitaxial MgB2 films fabricated by the *ex situ* annealing of chemical vapour deposition-grown B films in Mg vapour. Supercond. Sci. Technol..

[43] Jung S-G, Seong WK, Kang WN (2013). Flux pinning mechanism in single-crystalline MgB_2_ thin films. J. Phys. Soc. Jpn..

[44] Seong WK, Oh S, Kang WN (2012). Perfect domain-lattice matching between MgB_2_ and Al2O3: Single-crystal MgB_2_ thin films grown on sapphire. Jpn. J. Appl. Phys..

