# Supplementary material for: Giant proximity effect in single-crystalline MgB2 bilayers
Source: Sci Rep. 2019 Mar 1;9:3315. doi: 10.1038/s41598-019-40263-9 (PMC6397212; doi:10.1038/s41598-019-40263-9)
Supplement: Supplementary file 1 — Supplementary Information [file 41598_2019_40263_MOESM1_ESM.docx]

**Supplementary Information for “Giant proximity effect in single-crystalline MgB_2_ bilayers”**

**Soon-Gil Jung^1,2^, Duong Pham**^2^**, Tae-Ho Park**^2^**, Han-Yong Choi**^2^**, Jin Won Seo**^3^**, Won Nam Kang**^2,*^**, and Tuson Park**^1,2,^**^†^**

^1^*Center for Quantum Materials and Superconductivity (CQMS), Sungkyunkwan University, Suwon 16419, Republic of Korea*

^2^*Department of Physics, Sungkyunkwan University, Suwon 16419, Republic of Korea*

^3^*Department of Materials Engineering, KU Leuven, Kasteelpark Arenberg 44-bus 2450, B-3001 Leuven, Belgium*

*Corresponding authors: Tuson Park (e-mail: [tp8701@skku.edu](mailto:tp8701@skku.edu)), Won Nam Kang (e-mail: [wnkang@skku.edu](mailto:wnkang@skku.edu))

**In this supplement, we describe methods and show additional data and analyses that support the results in the main text.**

**Figure S1**. Temperature dependence of resistivity (*ρ*) for (a) pristine and (b) Co-ion (1 × 10^14^ atoms/cm^2^) irradiated MgB_2_ thin films, where the resistivity is normalized at the resistivity value at 41 K, *ρ*(41 K) for comparison. The large residual resistivity ratio (RRR) of pristine MgB_2_ films indicates high quality of films [S1, S2], which was largely suppressed after Co-ion irradiation. Inset of Fig. S1(b) shows the ratio of RRR value of pristine (RRR_pri._) and irradiated (RRR_irr._) MgB_2_ films. In spite of a significant difference of RRR value of each films, the similar ratio of RRR_pri._/RRR_irr._ implies that the effect of irradiated Co ions is similar regardless of the thickness of films.

**Figure S2**. Zero-field-cooled (ZFC) and field-cooled (FC) dc magnetization (*M*) before and after 140 keV Co-ion irradiation with a dose of 1 × 10^14^ Co atoms/cm^2^. (a) Temperature dependences of normalized *M*(*T*) for pristine MgB_2_ films with thickness of 130 (MB130nm), 410 (MB410nm), 850 (MB850nm), and 1,300 nm (MB1300nm), where *M*(*T*) is normalized to the absolute ZFC *M* value at 5 K for comparison. A little broad transition in the MB130nm compared to the others is due to the thickness. (b) and (c) *M*(*T*) curves for the pristine (pri.) and the irradiated (irr.) MB130nm and MB410nm, respectively. Superconductivity of MB1300nm is significantly suppressed after Co-ion irradiation because the thickness of MB130nm is similar to the mean projected range (*δ* = 95 nm) of irradiated C ions. MB410nm also shows a large decrease of transition temperature of ZFC magnetization, even though the thickness of undamaged MgB_2_ layer is sufficiently thick, indicating a possibility of large inverse proximity effect. (d) Normalized *M*(*T*) curves for MB850nm and MB1300nm before and after irradiations.

**Figure S3**. Magnetization hysteresis (*M – H*) loops at 5 K for the pristine and 140 keV Co-ion irradiated MB410nm, where the *M – H* loops were measured from -1 T and magnetic fields were applied perpendicular to the film’s plane (*H//c*). The magnetic field dependence of critical current density, *J*_c_(*H*), was estimated from the *M – H* loops by using Bean’s critical state model (*J*_c_ = 30Δ*M/d*) [S3]. Here, Δ*M* (=Δ*m/V*) is the difference of magnetization value at the same magnetic field in the irreversible regions, as indicated in Fig. S3, and *V* (= *A* × *t*) is the volume of film, *t* is the thickness of film, and *d* is the corresponding diameter of the total area, *A =* π(*d*/2)^2^, of the film’s surface. The *J*_c_(*H*) for all MgB_2_ films presented in this study was determined from this manner.

**Supplementary references**

[S1] Xi, X. X., Pogrebnyakov, A. V., Xu, S. Y., Chen, K., Cui, Y., Maertz, E. C., Zhuang, C. G., Li, Q., Lamborn, D. R., Redwing, J. M., Liu, Z. K., Soukiassian, A., Schlom, D. G., Weng, X. J., Dickey, E. C., Chen, Y. B., Tian, W., Pan, X. Q., Cybart, S. A. & Dynes, R. C. MgB_2_ thin films by hybrid physical–chemical vapor deposition, *Physica C* **456**, 22 (2007).

[S2] Pogrebnyakov, A. V., Redwing, J. M., Jones, J. E., Xi, X. X., Xu, S. Y., Li, Q., Vaithyanathan, V. & Schlom, D. G. Thickness dependence of the properties of epitaxial MgB_2_ thin films grown by hybrid physical-chemical vapor deposition, *Appl. Phys. Lett.* **82**, 4319 (2003).

[S3] Kim, H. -J., Kang, W. N., Choi, E. -M., Kim, M. -S, Kim, K. H. P. & Lee, S. –I. High current-carrying capability in *c*-axis-oriented superconducting MgB_2_ thin films. *Phys. Rev. Lett.* **87**, 087002 (2001).
